# Diagnostic Utility of Minor Salivary Gland Biopsy for Primary Sjögren Syndrome in Patients With Negative Anti-SSA Antibodies

**DOI:** 10.7759/cureus.46207

**Published:** 2023-09-29

**Authors:** Rohit R Goel, Mark Jeranko, Lamont Jones, Amita Bishnoi, Alireza Meysami

**Affiliations:** 1 Rheumatology, Henry Ford Health System, Detroit, USA; 2 Rheumatology, Colorado Center for Arthritis and Osteoporosis, Englewood, USA; 3 Otolaryngology, Henry Ford Health System, Detroit, USA

**Keywords:** extraglandular manifestation, sialadenitis, focus score, dry mouth, dry eye, american college of rheumatology (acr), anti-ssa antibody, sicca, minor salivary gland biopsy, sjögren syndrome

## Abstract

Background: Sjögren syndrome is a systemic autoimmune disease characterized by lacrimal and salivary gland inflammation resulting in dry eyes and mouth. Although it is a common disease, diagnosis can be challenging due to its heterogeneous presentation. A positive minor salivary gland biopsy is mandatory to fulfill the 2016 American College of Rheumatology/European League Against Rheumatism (ACR/EULAR) classification criteria for primary Sjögren syndrome in patients who are seronegative for anti-SSA/Ro antibodies. The objective of our study was to evaluate the validity of minor salivary gland biopsy for patients who are SSA antibody-negative yet are suspected of having primary Sjögren syndrome because of compelling symptoms.

Methods: We conducted a retrospective chart review of adult patients with a negative anti-SSA antibody test who underwent minor salivary gland biopsy to assess suspected Sjögren syndrome at Henry Ford Rheumatology Clinics between January 2005 and December 2019. Patient characteristics and clinical features are described. Sensitivity, specificity, positive predictive value, and negative predictive value are assessed.

Results: A total of 47 patients were included: 46 (97.9%) females and one (2.1%) male. The mean age was 57.2 ± 13.8 years. There were 14 (29.8%) patients who had a positive minor salivary gland biopsy result and 15 (31.9%) patients who had a final diagnosis of Sjögren syndrome. Minor salivary gland biopsy had 93.3% sensitivity (95% confidence interval (CI): 68%-99.8%), 100% specificity (95% CI: 89.1%-100%), 100% positive predictive value (95% CI: 76.8%-100%), and 97% negative predictive value (95% CI: 84.2%-99.9%).

Conclusion: The diagnostic value of minor salivary gland biopsy is high for patients who do not have anti-SSA antibodies yet are suspected of having Sjögren syndrome. The results of the study support the consideration of routine minor salivary gland biopsy for identifying Sjögren syndrome in these patients.

## Introduction

Sjögren syndrome is a systemic autoimmune disease characterized by chronic infiltration of T-lymphocytes and B-lymphocytes in the lacrimal and salivary glands [1,2]. Affecting primarily females between the ages of 30 and 50 years old with a female-to-male ratio of 9:1, Sjögren syndrome is estimated to affect approximately 0.5% of the total population [3]. The production of inflammatory cytokines leads to glandular dysfunction resulting in dry eyes and mouth. Numerous extraglandular features may also be present [4-6]. Organ systems that may be involved in Sjögren syndrome include the skin and joints, as well as cardiovascular, pulmonary, gastrointestinal, renal, urologic, and peripheral and central nervous systems. Hematologic abnormalities can also occur, notably an increased risk for the development of non-Hodgkin lymphoma [7].

Sjögren syndrome is classified as primary when it is not associated with another autoimmune disease and secondary when associated with another autoimmune disease [8]. The diagnosis of Sjögren syndrome can be challenging due to its heterogeneous presentation. Diagnoses can be made in individuals with objective evidence of oral dryness, ocular dryness, glandular damage, and evidence of underlying autoimmunity. Symptoms that should lead to the suspicion of Sjögren syndrome include persistent dry eyes, dry mouth, parotid gland enlargement, and an unexplained increase in dental caries. Abnormal serological test results such as positive anti-SSA antibodies, positive rheumatoid factor, and hyperglobulinemia may also be seen [9]. Given the lack of a single diagnostic test for Sjögren syndrome, clinical diagnoses are generally made based on compatible clinical and laboratory features after the exclusion of alternative causes for dry eyes and dry mouth. Minor salivary gland biopsy is being widely accepted as the gold standard for diagnosis of Sjögren syndrome [10]. A positive minor salivary gland biopsy is mandatory to fulfill the 2016 American College of Rheumatology/European League Against Rheumatism (ACR/EULAR) classification criteria for primary Sjögren syndrome for patients who are seronegative for anti-SSA/Ro antibodies and have evidence of glandular dysfunction [11]. However, few validity or reliability studies have been done to verify the diagnostic utility of minor salivary gland biopsy for accurate diagnosis of Sjögren syndrome in this specific patient population [12]. Therefore, the objective of our retrospective study was to evaluate the diagnostic validity of minor salivary gland biopsy in patients who have had negative anti-SSA test results and yet were nonetheless suspected of having primary Sjögren syndrome because of compelling symptoms. We hypothesized that a minor salivary gland biopsy for determining Sjögren syndrome in this group of patients would have low diagnostic validity. Determining the real-world patterns of results from various Sjögren syndrome diagnostic strategies may help support or refute the current practice of routine minor salivary gland biopsy for patients with symptoms of Sjögren syndrome within the context of negative clinical indicators.

## Materials and methods

A retrospective medical chart review was conducted of patients who had negative anti-SSA antibody test results and underwent minor salivary gland biopsy as part of a workup for the diagnosis of possible Sjögren syndrome in the Department of Rheumatology at Henry Ford Health between January 2005 and December 2019. All patients had at least one symptom of ocular or oral dryness suggestive of possible Sjögren syndrome, which led to the initiation of biopsy testing. Ocular or oral dryness was defined as a positive response to at least one of the following questions: (1) Have you had daily, persistent, troublesome dry eyes for more than three months, (2) do you have a recurrent sensation of sand or gravel in the eyes, (3) do you use tear substitutes more than three times a day, (4) have you had a daily feeling of dry mouth for more than three months, and (5) do you frequently drink liquids to aid in swallowing dry food? Patients with any of the following were excluded: a history of head and neck radiation treatment, active hepatitis C infection, HIV/AIDS, sarcoidosis, amyloidosis, graft-versus-host disease, or immunoglobulin G4-related disease. Patients were identified by medical record number, and clinical characteristics were documented in a spreadsheet for statistical analysis. Patients were at least 18 years old and thought to have Sjögren syndrome based on symptoms. Clinical and demographic characteristics were extracted, including age, sex, presence of sicca symptoms only, systemic symptoms only, systemic and sicca symptoms, antinuclear antibody (ANA), rheumatoid factor, anti-SSB antibody, anticholinergic medication use, type 2 diabetes, thyroid disease, current tobacco use, ocular staining score ≥ 5, and Schirmer’s test ≤ 5 mm/five minutes. The primary outcomes of the study were a positive minor salivary gland biopsy test and a diagnosis of Sjögren syndrome. A positive minor salivary gland biopsy result was defined as a focus score of ≥1 per the pathologist’s interpretation. A diagnosis of Sjögren syndrome was made based on the rheumatologist’s opinion or score of ≥4 per the 2016 ACR/EULAR criteria.

Statistical analysis

Continuous variables were reported as mean and standard deviation (SD), and categorical variables were reported as count and frequency. Sensitivity, specificity, positive predictive value, and negative predictive value estimates along with corresponding 95% confidence intervals (CIs) were determined to assess the ability of the salivary gland biopsy to predict the diagnosis of Sjögren syndrome. The testing level for all comparisons was 0.05. All analyses were performed in Statistical Analysis System (SAS) 9.4 (SAS Institute Inc., Cary, NC, USA).

Ethical disclosure

Because this was a retrospective chart review, there were no known anticipated risks to participants, society, or privacy concerns. There were no anticipated direct benefits to the participants in the study. The anticipated risk was low, and the benefit was high. Participants did not receive compensation. The only cost associated with the research study was the statistical analysis. To minimize risks of breach of confidentiality, patient information was deidentified and listed with only the medical record number and kept in a secure spreadsheet. Permission to conduct this study was granted by the Henry Ford Health System Institutional Review Board (number 13354).

## Results

A total of 47 patients were included in the analysis, and almost all were females (46 (97.9%) females and one (2.1%) male). The mean age for the entire group was 57.2 ± 13.8 years. Only 14 (29.8%) patients were on anticholinergic therapy, and only two (4.3%) reported current tobacco use. Regarding symptoms potentially indicative of Sjögren syndrome, 11 (23.4%) had sicca symptoms only, one (2.1%) had systemic symptoms only, and 35 (74.5%) had both. Only one (2.1%) patient had type 2 diabetes, and nine (19.1%) had thyroid disease (Table 1).

**Table 1:** Baseline characteristics of patients with negative anti-SSA antibodies undergoing minor salivary gland biopsy for suspected Sjögren syndrome ANA: antinuclear antibody, RF: rheumatoid factor, SD: standard deviation *Some patients did not have certain tests done; row percentages are reported.

Characteristics	Number (%) (N=47)
Sex	
Female	46 (97.9)
Male	1 (2.1)
Age, years (mean ± SD)	57.2 ± 13.8
Tobacco use	2 (4.3)
Anticholinergic medical use	14 (29.8)
Systemic and sicca symptoms	
Sicca symptoms only	11 (23.4)
Systemic symptoms only	1 (2.1)
Systemic and sicca symptoms	35 (74.5)
Comorbidities	
Type 2 diabetes	1 (2.1)
Thyroid disease	9 (19.1)
Clinical test results	
Anti-SSB antibody positive	5 (10.6)
^*^ANA positive (n=44)	30/44 (68.2)
^*^RF positive (n=45)	4/45 (8.9)
^*^Schirmer’s test ≤ 5 mm/5 minutes (n=11)	6/11 (54.5)
Main outcomes	
Minor salivary gland biopsy positive	14 (29.8)
Diagnosis of Sjögren syndrome	15 (31.9)

Patients had a variety of laboratory tests performed. Only five (10.6%) patients had a positive anti-SSB antibody test result. Of the 44 patients who had an ANA test, 30 (68.2%) had a positive result. Of the 45 patients who had rheumatoid factor assessed, only four (8.5%) had a positive result. Of the 11 patients who had a Schirmer’s test done, six (54.5%) had a positive result (Table 1).

There were 14 (29.8%) patients who had a positive minor salivary gland biopsy result and 15 (31.9%) patients who had a final positive diagnosis of Sjögren syndrome (Table 1). Validity testing revealed that minor salivary gland biopsy had a 93.3% sensitivity (95% CI: 68%-99.8%), 100% specificity (95% CI: 89.1%-100%), 100% positive predictive value (95% CI: 76.8%-100%), and 97% negative predictive value (95% CI: 84.2%-99.9%) in this patient cohort (Table 2).

**Table 2:** Sensitivity, specificity, predictive values, and corresponding confidence intervals

Statistic	X/N	Probability	95% confidence interval
Sensitivity	14/15	93.3%	68%-99.8%
Specificity	32/32	100%	89.1%-100%
Positive predictive value	14/14	100%	76.8%-100%
Negative predictive value	32/33	97%	84.2%-99.9%

## Discussion

Sjögren syndrome should be suspected in a patient with daily ocular and oral dryness for three or more months. Workup should begin with a thorough medical history and physical examination. Diagnostic testing includes laboratory testing, ophthalmologic examination, and evaluation for salivary hypofunction. Labial salivary gland biopsy is an important diagnostic test in patients who lack evidence of systemic autoimmunity such as anti-SSA antibodies or a concomitant autoimmune disease. The potential benefit of our findings to the rheumatology community is that routine minor salivary gland biopsy for assessing Sjögren syndrome in patients with negative anti-SSA antibodies may, indeed, be supported. Data refuting the reliability of routine biopsy could require modification of our current practice to expose fewer patients to the potential harm from an invasive procedure.

In this retrospective study exploring the diagnostic utility of minor salivary gland biopsy for patients who are suspected of having primary Sjögren syndrome but who have had negative anti-SSA antibody test results, we observed that approximately 30% of patients had a positive biopsy result, and about 32% ultimately received a diagnosis of Sjögren syndrome. Our findings indicate that a glandular biopsy diagnostic approach has high specificity and good predictive value, indicating clinical usefulness for determining Sjögren syndrome in patients with negative laboratory indicators.

Sicca symptoms and certain extraglandular symptoms of Sjögren syndrome by themselves are nonspecific [9]. Per the 2016 classification criteria update, positive serology for anti-SSB/La antibodies in the absence of anti-SSA/Ro antibodies is no longer considered a criteria item. This guideline modification was based on expert consensus opinion and a study that showed no significant association between Sjögren syndrome phenotypic features and anti-SSB/La seronegativity [13]. The 2016 ACR/EULAR criteria for primary Sjögren syndrome applies to individuals who have at least one symptom of ocular or oral dryness, do not have any condition listed as exclusion criteria (a history of head/neck radiation, active hepatitis C infection, AIDS, sarcoidosis, amyloidosis, graft-versus-host disease, or immunoglobulin G4-related disease), and have a score of ≥4 when the weights from the five following criteria are summed: labial salivary gland with focal lymphocytic sialadenitis and focus score of ≥1 foci/4 mm^2^ (3 points), anti-SSA antibody positivity (3 points), ocular staining score ≥ 5 in at least one eye (1 point), Schirmer’s test ≤ 5 mm/five minutes in at least one eye (1 point), and unstimulated whole saliva flow rate ≤ 0.1 mL/minute (1 point).

A positive minor salivary gland biopsy result is required for a definitive diagnosis of primary Sjögren syndrome in patients who lack anti-SSA antibodies. Few studies have been done to explore the diagnostic validity or reliability of minor salivary gland biopsy in this specific patient population. Advantages of minor salivary gland biopsy include confirmation of a suspected diagnosis of Sjögren syndrome and exclusion of other causes of salivary hypofunction and bilateral gland enlargement. Rarely, lymphoma may be revealed, and biopsy can provide valuable prognostic information [14]. For biopsy results to be considered acceptable for evaluation, at least four lobules of salivary gland tissue should be obtained [15]. A focus is defined as 50 or more mononuclear cells/mm^2^. Guidelines for interpreting labial salivary gland biopsies are being established, and experience in reading them is critical for proper interpretation [16].

While the labial salivary gland biopsy is an important diagnostic tool for Sjögren syndrome, there is a risk associated with the procedure. Up to 6% of patients will develop persistent lip numbness due to damage to the sensory nerves beneath the salivary gland [17]. However, this complication can be minimized by using minimally invasive techniques [18,19].

Interestingly, one patient in our study was given a diagnosis of Sjögren syndrome despite having had a negative minor salivary gland biopsy result. The patient initially presented with dry eyes, dry mouth, and inflammatory arthritis, with positive anti-ANA and rheumatoid factor antibodies. A Schirmer’s test was performed, which was 4 mm on the right and 3 mm on the left, indicating a positive result. The minor salivary gland biopsy that was performed showed minimal, chronic sialadenitis. Five lobules were included, which was well within the recommended range of 4-7. The patient’s focus score, as defined by Greenspan et al. [20], was 0 foci/4 mm^2^, and the Tarpley grade of the biopsy was zero. Tarpley grades of 4 or more are only seen in patients with Sjögren syndrome, and grades of 2 or 3 are considered consistent with this disorder [21]. Although the patient’s histologic findings did not support a diagnosis of Sjögren syndrome, the diagnosis could not be definitively ruled out because of the possibility of sampling error.

Limitations of our study include a small sample size. Given that the study was retrospective in nature, there was a higher risk for bias. While Sjögren syndrome does have a strong female predisposition, only one patient in our study was male. One of the patients in the study was given a diagnosis of Sjögren despite having had a negative minor salivary gland biopsy and not fulfilling the 2016 ACR/EULAR criteria. However, the diagnosis was made based on an expert rheumatologist’s opinion, which can be considered less objective.

## Conclusions

The diagnostic value of minor salivary gland biopsy is high for patients who do not have anti-SSA antibodies but are suspected of having Sjögren syndrome based on compelling symptoms. We observed that 14 of 47 (29.8%) patients who underwent minor salivary gland biopsy had a positive result. The benefit of minor salivary gland biopsy outweighs the inherent risk given the useful diagnostic information that can be obtained from the procedure. The results of this study support consideration of routine minor salivary gland biopsy for patients exhibiting Sjögren syndrome symptoms in the absence of anti-SSA antibodies.
